# Reply to the Letter to the Editor “Considerations on allergic rhinitis associated with the degree of pulmonary involvement due to COVID-19 in patients from a Peruvian general hospital”

**DOI:** 10.17843/rpmesp.2023.402.12959

**Published:** 2023-06-30

**Authors:** Bianca García-Gallo, Giancarlo Gonzales-Caldas, Diego Urrunaga-Pastor, Percy Herrera-Añazco

**Affiliations:** 1 Universidad Peruana de Ciencias Aplicadas, Lima, Peru. Universidad Peruana de Ciencias Aplicadas Universidad Peruana de Ciencias Aplicadas Lima Peru; 2 Universidad Científica del Sur, Lima, Peru. Universidad Científica del Sur Universidad Científica del Sur Lima Peru; 3 Universidad Privada del Norte, Trujillo, Peru. Universidad Privada del Norte Universidad Privada del Norte Trujillo Peru

To the editor. We thank the authors of the letter for the interest and time dedicated to reviewing our article, which aimed to evaluate the association between the presence of allergic rhinitis and the degree of pulmonary involvement in patients with COVID-19. This research, with results that we consider to be preliminary, found that the history of allergic rhinitis represented a decrease of 30% in the severity of COVID-19 in hospitalized patients according to the tomographic score (TS) ^1^.

Some limitations are mentioned in the manuscript, like the fact that more rigorous studies were needed to explain this possible association. However, we agree that some limitations could have been described in more detail, as the authors of the letter have pointed out. This includes other comorbidities that were not considered in our study that could potentially increase the risk of severe pulmonary involvement in patients with COVID-19 as well as having a more detailed description of the history of allergic rhinitis. Unfortunately, as was also pointed out in our article, the information and variables that were included in the study were those that were found in the medical records, therefore, we were not able to include other variables that may have contributed to explain the association. Besides, although these variables could be other respiratory parameters as the authors pointed out, the increase of TS is associated with increased mortality, disease severity and can predict hospital stay and the need for intubation of infected patients ^1^. Thus, we consider that this variable could be relevant for our research, in which we sought to estimate, in an exploratory manner, an association of interest.

We found no evidence that the history of vaccination against COVID-19 could modify the association between the history of allergic rhinitis and the degree of pulmonary involvement in patients with COVID-19. However, the history of vaccination against COVID-19 does decrease the risk of severity ^2^ and thus could modify the association of interest between a history of allergic rhinitis and the TS, for which reason we agree that it should have been included in the study. However, since vaccination in Peru began in February 2021 ^3^ and the fact that our study included patients from 2020, the sample would probably not have been sufficient to determine the true effect of vaccination.

As the authors pointed out, there is controversy regarding the association of interest that is described in our study; and as we mentioned in our manuscript, we consider that the topic merits the development of studies with better methodological quality.
