# Supplementary material for: Reply to the Letter to the Editor “Considerations on allergic rhinitis associated with the degree of pulmonary involvement due to COVID-19 in patients from a Peruvian general hospital”
Source: Rev Peru Med Exp Salud Publica. 2023 Jun 30;40(2):252. doi: 10.17843/rpmesp.2023.402.12959 (PMC11061879; doi:10.17843/rpmesp.2023.402.12959)
Supplement: Translation

# Translation: Réplica a la Carta al Editor “Consideraciones sobre la rinitis alérgica asociada al grado de compromiso pulmonar por COVID-19 en pacientes de un hospital general peruano”

**Authors:** Bianca García-Gallo, Giancarlo Gonzales-Caldas, Diego Urrunaga-Pastor, Percy Herrera-Añazco

**Affiliations:** 1 Universidad Peruana de Ciencias Aplicadas, Lima, Perú.; 2 Universidad Científica del Sur, Lima, Perú.; 3 Universidad Privada del Norte, Trujillo, Perú.

Sr. Editor, agradecemos a los autores de la carta, el interés y el tiempo dedicado a la revisión de nuestra investigación que tuvo como objetivo evaluar la asociación entre la presencia de rinitis alérgica y el grado de compromiso pulmonar en pacientes con COVID-19. Esta investigación, con resultados que calificamos como preliminares, encontró que el antecedente de rinitis alérgica representó una disminución en la gravedad de COVID-19 según el puntaje tomográfico (PT) del 30,0% en pacientes hospitalizados ^1^.

En el manuscrito se resaltan algunas limitaciones que nos obligaron a señalar que, eran necesarios estudios más rigurosos para dilucidar esta posible asociación. Sin embargo, estamos de acuerdo en que algunas limitaciones podrían mencionarse con más detalles como los autores de la carta han señalado. Eso incluye, efectivamente, incluir otras comorbilidades que no fueron consideradas en nuestro estudio y aumentan, potencialmente, el riesgo de compromiso pulmonar severo en los pacientes con COVID-19 y, como también señalamos, mayor detalle en la descripción del antecedente de rinitis alérgica. Desafortunadamente, como también fue señalado en nuestro estudio, la información y variables incluidas en el estudio correspondía a la registrada en las historias clínicas y pudo no incluir variables relevantes para explicar la asociación de interés. Asimismo, pese a que entre estas variables podrían hallarse otros parámetros respiratorios como señalaron los autores, el PT es una variable cuyo aumento está asociado con el incremento de la mortalidad, severidad de la enfermedad y predice la estancia hospitalaria y necesidad de intubación en los pacientes infectados ^1^. Así, consideramos que esta variable podría ser relevante para el presente estudio de investigación que buscó estimar de manera exploratoria una asociación de interés.

Con respecto al antecedente de vacunación contra la COVID-19, no hallamos evidencia que indique que esta variable pueda modificar la asociación entre el antecedente de rinitis alérgica y el grado de compromiso pulmonar en pacientes con COVID-19. No obstante, el antecedente de vacunación contra la COVID-19 disminuye el riesgo de severidad ^2^ y así, podría representar una variable que modificaría la asociación de interés entre el antecedente de rinitis alérgica y el PT, por lo cual, estamos de acuerdo en que debió ser incluida en el estudio. No obstante, debido a que la vacunación en el Perú empezó en febrero de 2021 ^3^^)^ y nuestro estudio incluyó pacientes desde el año 2020, probablemente la muestra no hubiera sido suficiente para determinar el verdadero efecto de la vacunación en nuestros resultados.

Como los autores señalan, existe controversia con respecto a la asociación de interés descrita en nuestro estudio, y tal como señalamos en nuestro manuscrito, consideramos que el tópico amerita el desarrollo de estudios con una mejor calidad metodológica.
